# Supplementary material for: Tuning the many-body interactions in a helical Luttinger liquid
Source: Nat Commun. 2022 Oct 20;13:6046. doi: 10.1038/s41467-022-33676-0 (PMC9584911; doi:10.1038/s41467-022-33676-0)
Supplement: Supplementary file 1 — Supplementary Information [file 41467_2022_33676_MOESM1_ESM.pdf]

Supplementary Information for:
Tuning the Many-body Interactions in a Helical Luttinger Liquid

Junxiang Jia,[1] Elizabeth Marcellina,[1] Anirban Das,[2,3] Michael S. Lodge,[1] BaoKai Wang,[4] Duc Quan Ho,[1] Riddhi Biswas,[1] Tuan Anh Pham,[1] Wei Tao,[1] Cheng-Yi Huang,[4] Hsin Lin,[5] Arun Bansil,[4] Shantanu Mukherjee,[2,3,6] and Bent Weber[1,7,*]

[1]*Division of Physics and Applied Physics, School of Physical and Mathematical Sciences, Nanyang Technological University, Singapore 637371, Singapore*
[2]*Department of Physics, Indian Institute Of Technology Madras, Chennai, Tamil Nadu 600036, India*
[3]*Center for Atomistic Modelling and Materials Design, Indian Institute Of Technology Madras, Chennai, Tamil Nadu 600036, India*
[4]*Department of Physics, Northeastern University, Boston, Massachusetts 02115, USA*
[5]*Institute of Physics, Academia Sinica, Taipei 115201, Taiwan*
[6]*Quantum Centre for Diamond and Emergent Materials, Indian Institute of Technology Madras, Chennai, Tamil Nadu 600036, India*
[7]*ARC Centre of Excellence for Future Low-Energy Electronics Technologies (FLEET), School of Physics & Astronomy, Monash University, Clayton 3800, Australia*
(Dated: September 20, 2022)

Contents

S1. MBE growth and Differential Conductance Mapping 1

S2. Temperature-Dependent Scanning Tunnelling Spectroscopy of the TLL 2

S3. The dependence of the ZBA conductance on the lock-in amplitude 3

S4. Ruling out a Coulomb gap as origin of the zero bias anomaly 4

S5. Statistical distributions for K and α 6

S6. Electrostatic model to estimate the Luttinger parameter K 7

S7. Full tight-binding model to calculate the Luttinger parameter K 8

References 11

S1. MBE GROWTH AND DIFFERENTIAL CONDUCTANCE MAPPING

Molecular-beam epitaxy (MBE) of monolayer 1T'-WTe_2 was carried out on highly oriented pyrolytic graphite (HOPG) and bilayer graphene (BLG) on 6H-SiC(0001) substrates. Prior to MBE, the HOPG substrates were mechanically cleaved in ambient conditions and then degassed at 300 ℃ overnight in ultrahigh vacuum (UHV) with a base pressure of 2×10^{-10} mbar. For the epitaxial growth of BLG, 6H-SiC(0001) substrates were degassed at 600 ℃ overnight in UHV and then flash annealed at 2000 ℃ for a few cycles. Monolayer 1T'-WTe_2 was synthesised thereafter in an Omicron Lab10 MBE system (base pressure: 2×10^{-10} mbar) with an electron beam evaporator (W source, purity = 99.998%) and a Knudsen diffusion cell (Te source, purity = 99.999%). We typically use a W:Te flux ratio of 1:280 and a substrate temperature of 165 ℃ for 2 hours to cover approximately 50% of the monolayer area.

A typical STM image of MBE-grown monolayer 1T'-WTe_2 island is shown in the left panel of Supplementary Fig. 1a (i.e., the same island as shown in Fig. 1a of the main text). The edges of the island are generally disordered, with edges parallel or perpendicular to the atomic rows. For all our measurements, we chose regions with clean surface and well-defined edges as seen in the lower half of the island. The middle and right panels of Supplementary Fig. 1a show the corresponding differential conductance $\mathrm{d}I/\mathrm{d}V$ maps of the whole island, recorded at energies -70 meV and -30 meV. At -70 meV, both the bulk and edge local density of states (LDOS) are enhanced compared to the

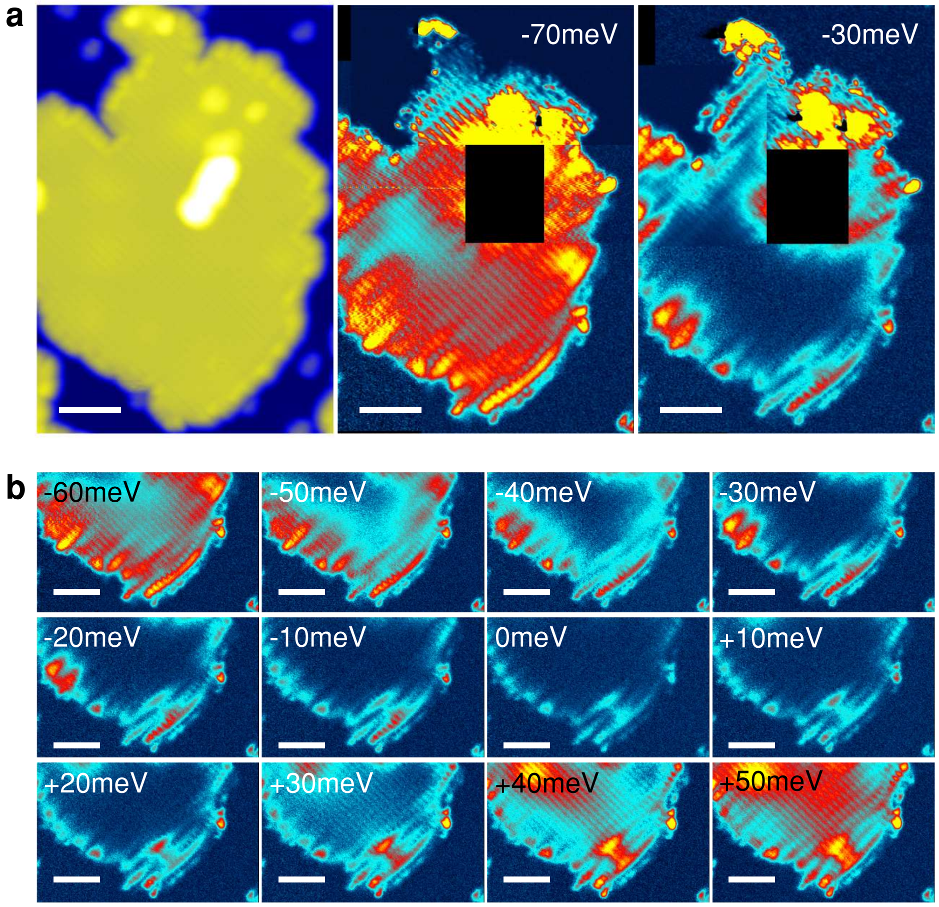

Supplementary Fig. 1: **STM topography image and differential conductance dI/dV maps of a monolayer 1T'-WTe_2.** **a** Topography image of a monolayer 1T'-WTe_2 island on HOPG (the same island in Fig. 1 of the main text) alongside its corresponding dI/dV maps at $E - E_F = -70$ meV and -30 meV (recorded in constant height mode). The black squares correspond to the sample regions omitted from the dI/dV scanning to avoid the impurities depicted by the white spots in **a**. **b,** dI/dV map of the impurity-free lower half of the island in **a**, measured at energies from $E - E_F = -60$ meV to $E - E_F = +50$ meV. An enhanced LDOS persists along the sample edge for all energies, but the bulk is insulating between approximately -40 meV and +20 meV, with the LDOS matching that of the HOPG substrate. All the scale bars in this figure represent 5 nm.

substrate LDOS, indicating a metallic 2D bulk and edge. At -30 meV, inside the bulk energy gap (see also the insert to Supplementary Fig. 1f), only the LDOS at the edge is enhanced while the bulk LDOS is strongly suppressed. The presence of edge states surrounding an insulating bulk is consistent with the expected signatures of a quantum spin Hall (QSH) insulator. Further dI/dV maps at different energies are shown in Supplementary Fig. 1b. We find that the enhanced edge state LDOS persist at all energies, while measurable bulk LDOS is only observed between $\simeq -40$ meV and $\simeq 30$ meV.

S2. TEMPERATURE-DEPENDENT SCANNING TUNNELLING SPECTROSCOPY OF THE TLL

The tunnelling conductance $\frac{dI}{dV}(E,T)$ at finite temperatures is modelled by the convolution of the TLL tunnelling density of states ρ_{TLL} and the first derivative of the Fermi-Dirac distribution $f(E)$, i.e.,

$$\frac{\mathrm{d}I}{\mathrm{d}V}(E,T) \propto \int_{-\infty}^{+\infty} \rho_{\mathrm{TLL}}(eV,T)\frac{\partial f(E-eV)}{\partial E}\mathrm{d}V, \tag{1}$$

where

$$\rho_{\mathrm{TLL}}(E,T) \propto T^{\alpha}\cosh\left(\frac{E}{2k_BT}\right)\left|\Gamma\left(\frac{1+\alpha}{2}+i\frac{E}{2\pi k_BT}\right)\right|^2, \tag{2}$$

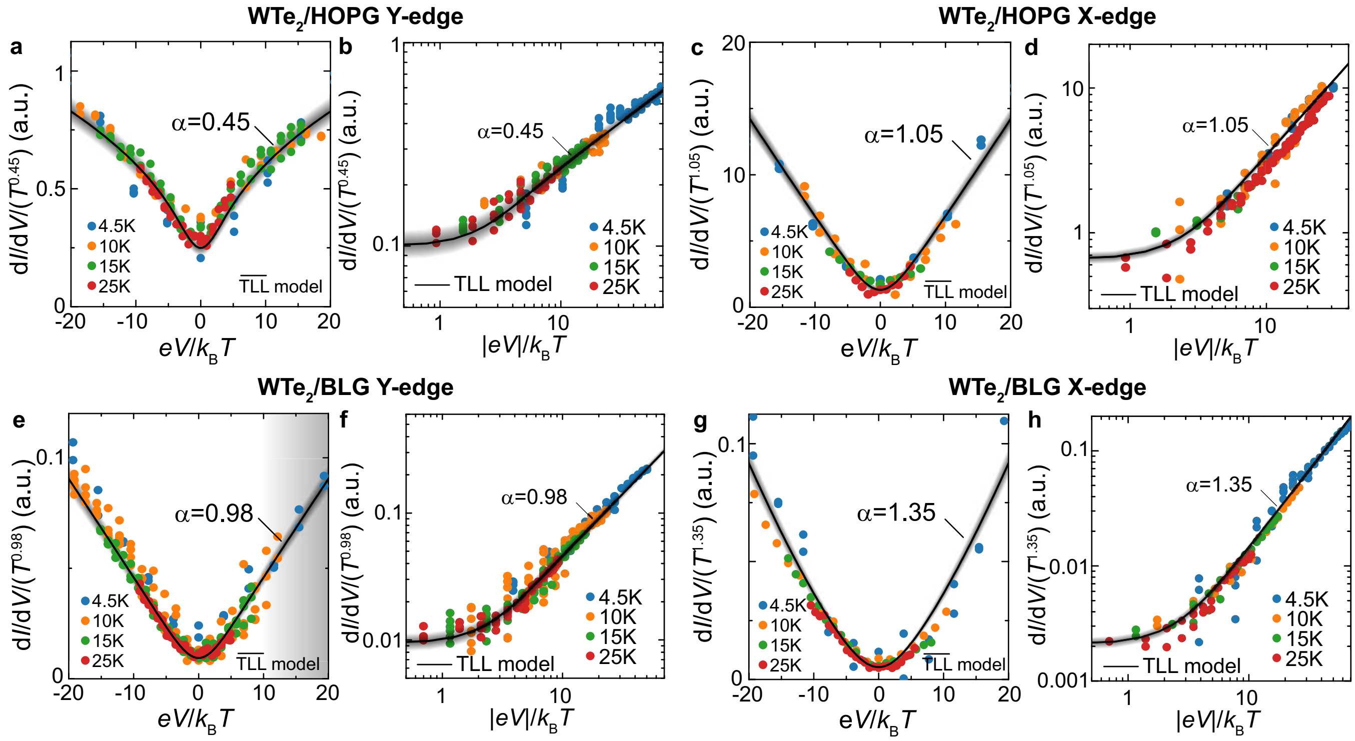

Supplementary Fig. 2: **Demonstration of universal scaling for the Y- and X- edges on HOPG and BLG substrates.** **a,c,e,g** Universal scaling for the Y- and X- edges on HOPG and BLG substrates. **b,d,f,h** Double-logarithmic plot of universal scaling. We find $\alpha = 0.45 \pm 0.15$ ($K = 0.40 \pm 0.05$) for WTe_2/HOPG Y-edge, $\alpha = 1.05 \pm 0.15$ ($K = 0.26 \pm 0.02$) for WTe_2/HOPG X-edge, $\alpha = 0.98 \pm 0.15$ ($K = 0.27 \pm 0.03$) for WTe_2/BLG Y-edge, and $\alpha = 1.35 \pm 0.15$ ($K = 0.22 \pm 0.01$) for WTe_2/BLG X-edge. All values extracted for K on the two different substrates and respective edges fall to within the normal distribution for K (Fig. 3 of the main text).

and $f(E) = 1/\{1 + \exp[(E - E_F)/(k_B T)]\}$. E_F is the Fermi energy, $E - E_F \equiv eV$ with the STM bias voltage V. α is the universal scaling exponent, e is the elementary charge, k_B is the Boltzmann constant, and T is the temperature. All the spectra were normalised at $E - E_F = -20$ meV and then fitted to Supplementary Equation (1).

Supplementary Fig. 2 shows a full set of data, demonstrating universal scaling for different edge terminations on both BLG and HOPG. For each substrate and edge, the tunnelling spectra collapse onto a single universal curve that fits to Supplementary Equation (2) with a common α. We find $\alpha = 0.45 \pm 0.15$ ($K = 0.40 \pm 0.05$) for WTe_2/HOPG Y-edge, $\alpha = 1.05 \pm 0.15$ ($K = 0.26 \pm 0.02$) for WTe_2/HOPG X-edge, $\alpha = 0.98 \pm 0.15$ ($K = 0.27 \pm 0.03$) for WTe_2/BLG Y-edge, and $\alpha = 1.35 \pm 0.15$ ($K = 0.22 \pm 0.01$) for WTe_2/BLG X-edge. All values extracted for K fall into the normal distributions shown in Fig. 3 of the main text.

S3. THE DEPENDENCE OF THE ZBA CONDUCTANCE ON THE LOCK-IN AMPLITUDE

All scanning tunnelling spectroscopy was carried out using standard lock-in techniques with a modulation amplitude of $V_{\rm lock-in} = 2$ mV. This modulation amplitude has been optimised to maximize the signal-to-noise ratio, while minimizing Gaussian broadening of the instrumental response. Supplementary Fig. 3a shows our amplitude calibration, comparing spectra of the TLL pseudogap, measured at $T = 4.5$ K and with lock-in amplitudes ranging from $V_{\rm lock-in} = 0.25$ mV to 10 mV. Spectra taken at $V_{\rm lock-in} < 3$ mV fit well to the TLL model with a common $\alpha = 0.83$ ($K = 0.3$). Instrumental broadening has a measurable effect only at $V_{\rm lock-in} > 3$ mV, more clearly seen in the zero-bias ($E = E_F$) tunneling conductance (Supplementary Fig. 3b). For spectra taken at $V_{\rm lock-in} < 1$ mV, we find that the signal-to-noise ratio substantially degrades. An AC modulation of 2 mV therefore provides a balance between optimized signal-to-noise ratio and suppressed instrumental broadening.

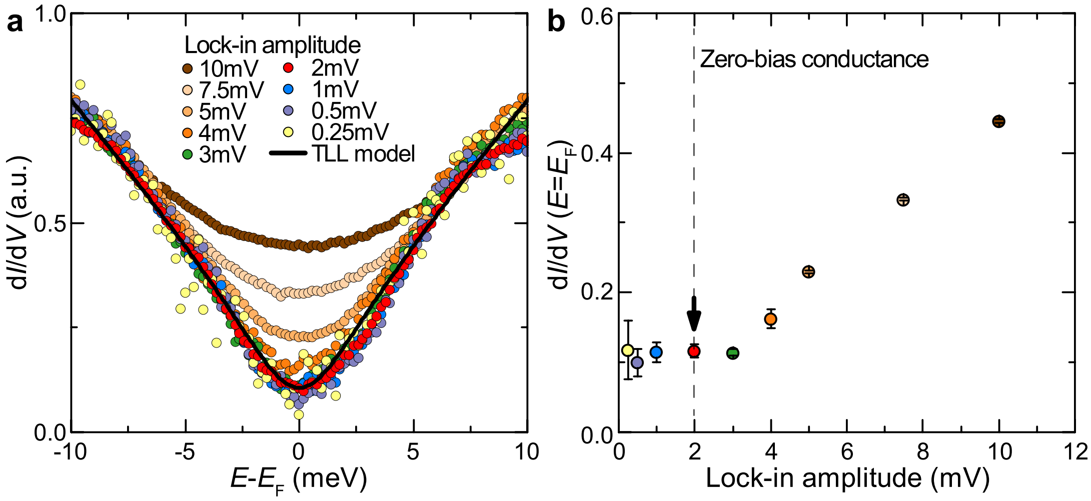

Supplementary Fig. 3: **Dependence of the TLL pseudogap on lock-in amplitude.** **a,** STS spectra of an edge of 1T'-WTe_2 on HOPG at $T = 4.5$ K taken with various lock-in amplitudes. **b,** The zero-bias conductance as a function of the lock-in amplitude, showing that the lock-in modulation amplitude of 2 mV provides the optimal compromise between high signal-to-noise ratio and low instrumental broadening.

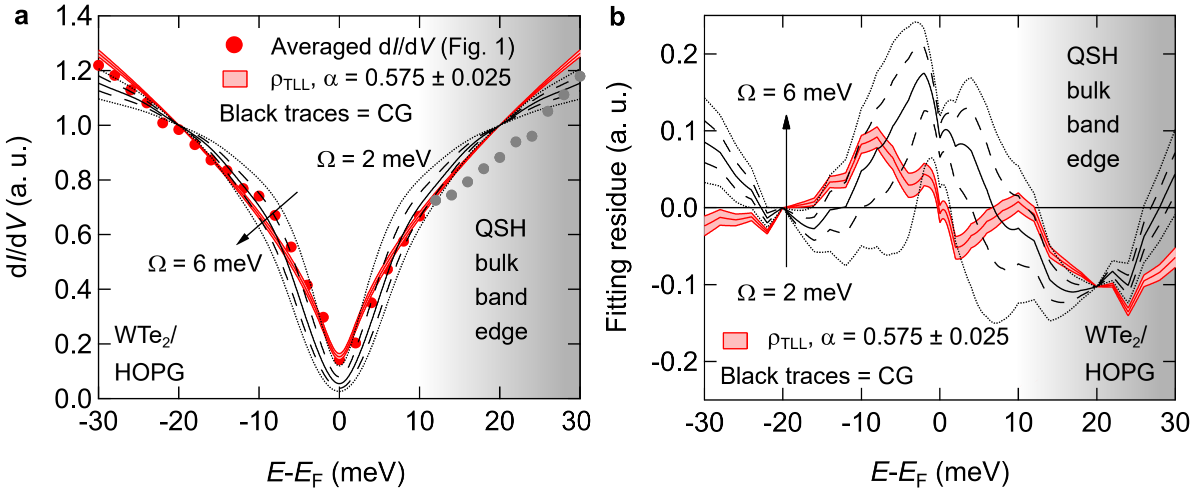

Supplementary Fig. 4: **Comparison of Tomonaga-Luttinger Liquid (TLL) and Coulomb gap (CG) models.** **a,** Spatially averaged low-noise spectrum acquired at the edge of the WTe_2 island shown in Fig. 1a of the main text. Black/grey solid and dashed lines are fits to the CG model of varying Ω, compared to our best fit to a TLL model ($\alpha = 0.575 \pm 0.025$). **b,** Fitting residue calculated for the traces in **a** to estimate the χ^2 value. We find acceptable fits to a CG model only for $\Omega = (4 \pm 2)$ meV. None of the CG fits can model the data over the full energy range, but selectively only at the ZBA minimum or its shoulder.

S4. RULING OUT A COULOMB GAP AS ORIGIN OF THE ZERO BIAS ANOMALY

An alternative mechanism, sometimes invoked to explain the presence of a zero bias anomaly (ZBA) is the formation of an Efros-Shklovskii Coulomb gap (CG) [1, 2], where long-range interactions between electrons cause the density of states (DOS) near the Fermi level to be suppressed. To rule out the presence of a CG, we fit the data in Fig. 2 of main text to the CG model. For the quasi-1D case, the DOS near the Fermi level is expressed as [3, 4]:

$$\rho_{\mathrm{CG,1D}}(E,T) = \rho_0 \coth\left(\frac{E}{2k_BT}\right)\left(\frac{2k_BT}{\hbar}\right)\int_0^\infty dt \frac{\sin\left(Et/\hbar\right)\cos\left(\sqrt{\Omega t/\hbar}\right)}{\sinh(\pi t k_{\mathrm{B}}T/\hbar)} \times \exp\left[-\sqrt{\frac{\Omega}{\pi}}\int_0^\infty \frac{1-\cos\left(E't/\hbar\right)}{E'^{3/2}\tanh\left(E'/2k_BT\right)}dE'\right] \tag{3}$$

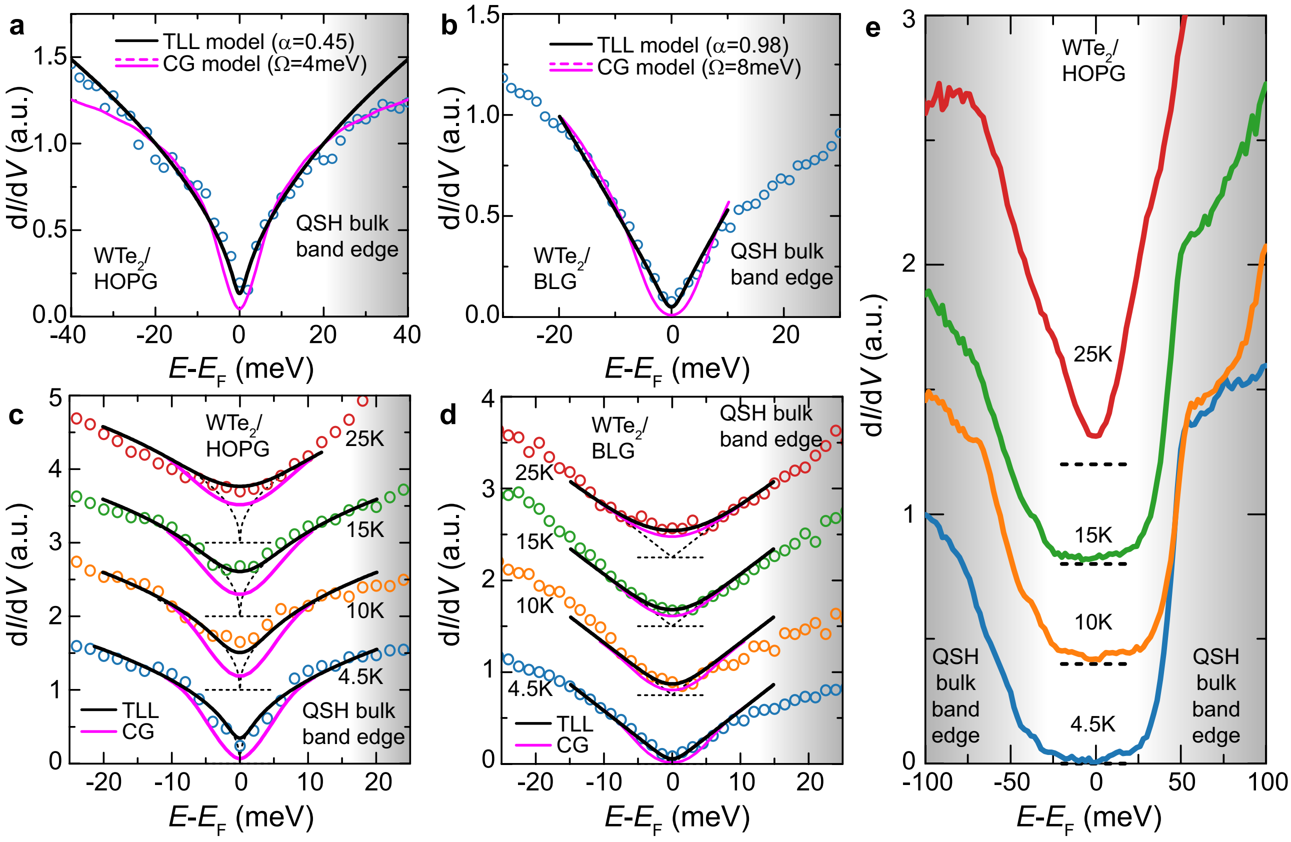

Supplementary Fig. 5: **Comparison of the Tomonaga-Luttinger Liquid (TLL) and Coulomb gap (CG) models for different temperatures. a,b** Typical dI/dV spectra of WTe_2 Y-edges on HOPG and BLG/SiC, respectively, taken at 4.5 K. **c,d** dI/dV spectra taken at temperatures between 4.5 K and 25 K. In **a-d**, the solid black and magenta lines represent the TLL and CG best fits (extracting $\Omega = 4$ meV for HOPG and $\Omega = 8$ meV for BLG, respectively). The comparison shows that the TLL model provides good agreement with the detailed functional form of the ZBA, while the CG model cannot describe the data well. **e,** Tunnelling spectra of the QSH bulk gap (WTe_2/HOPG) at different temperatures, stable up to $T \simeq 25$ K.

where E, E' represent energy, $\hbar$ is the reduced Planck constant, t is time, ρ_0 is a proportionality factor, and Ω is the frequency scale which is defined by:

$$\Omega = \frac{f_0^2}{32\pi\hbar D_0}. \tag{4}$$

Here, f_0 represents the effective electron-electron interaction potential corresponding to the long-range interactions between electrons. At $T \to 0$ K, f_0 is equal to the interaction potential $W(q = 0)$, only when the exchange part of the interactions is considered. D_0 is the diffusion coefficient. This makes Supplementary Equation (3) a single-parameter fit at each temperature with the frequency scale Ω the only variable.

Using the data presented in Fig. 1g of the main text, we illustrate how we differentiate between TLL and CG models. The same data, reproduced in Supplementary Fig. 4a, was spatially averaged to achieved improved noise levels and fit to Supplementary Equation (2) and (3), respectively. The corresponding fitting residues are shown in Supplementary Fig. 4b. We find that acceptable fits to a CG model can only be obtained for Ω values ranging between 2-6 meV. However, the fit can only reproduce, either, the minimum of the ZBA or the shape of its shoulder, but never both at the same time. In stark contrast the data well-described over the full energy range within the QSH gap by a TLL model with $\alpha = 0.575 \pm 0.025$. This is further reflected in a lower least squares value $\chi^2 = 1.75217$ for the TLL fit, compared to the best CG fit ($\chi^2 = 5.16056$ at $\Omega = 3$ meV for this data set). The same procedure has subsequently been applied to all universal scaling data sets on both HOPG and BLG substrates, including Supplementary Fig. 5a,b and Fig. 2e,f of the main text to determine the respective Ω at $T = 4.5$ K. The temperature dependence of the fits to both TLL (black lines) and CG models (magenta lines) between $4.5 \text{ K} \leq T \leq 25$ K are shown in Supplementary Fig. 5c,d. We find that the TLL model (black lines) provides better agreement with our experimental data for all

temperatures and both substrates, with the differences between CG and TLL most clearly visible in the semi-log plot of the zero-bias conductance shown in Fig. 2e,f of the main text. Correspondingly, Supplementary Fig. 5e shows the temperature dependence of the QSH bulk gap, which persists up to $T \approx 25$ K.

S5. STATISTICAL DISTRIBUTIONS FOR K AND α

The histograms in Supplementary Fig. 3a,b in the main text show the Luttinger parameter K extracted at different edges of the 1T'-WTe_2 crystal on HOPG and BLG substrates, respectively. A total of 69 locations were probed across different samples on both substrates and with 5 different tunneling tips. All four histograms fit well to Gaussian normal distributions, from which we extract mean and standard deviations for the Luttinger parameters K as listed in Table 1. The extracted values have subsequently been plotted as a function of substrate relative permittivity in Fig. 3c of the main text.

To further confirm normality of the four distributions, we produce Q-Q plots for all data sets, shown in Supplementary Fig. 6. For perfect normal distributions, the theoretically expected quantiles (which are obtained by taking the inverse of the cumulative distribution function of the Gaussian distribution) versus the raw data quantiles will lie on the identity line (red solid line) as shown. Slight skew (deviation of data quantiles from the identity line) is only observed for the X-edges of WTe_2/HOPG and WTe_2/BLG. Otherwise, the data is well described by normal distributions.

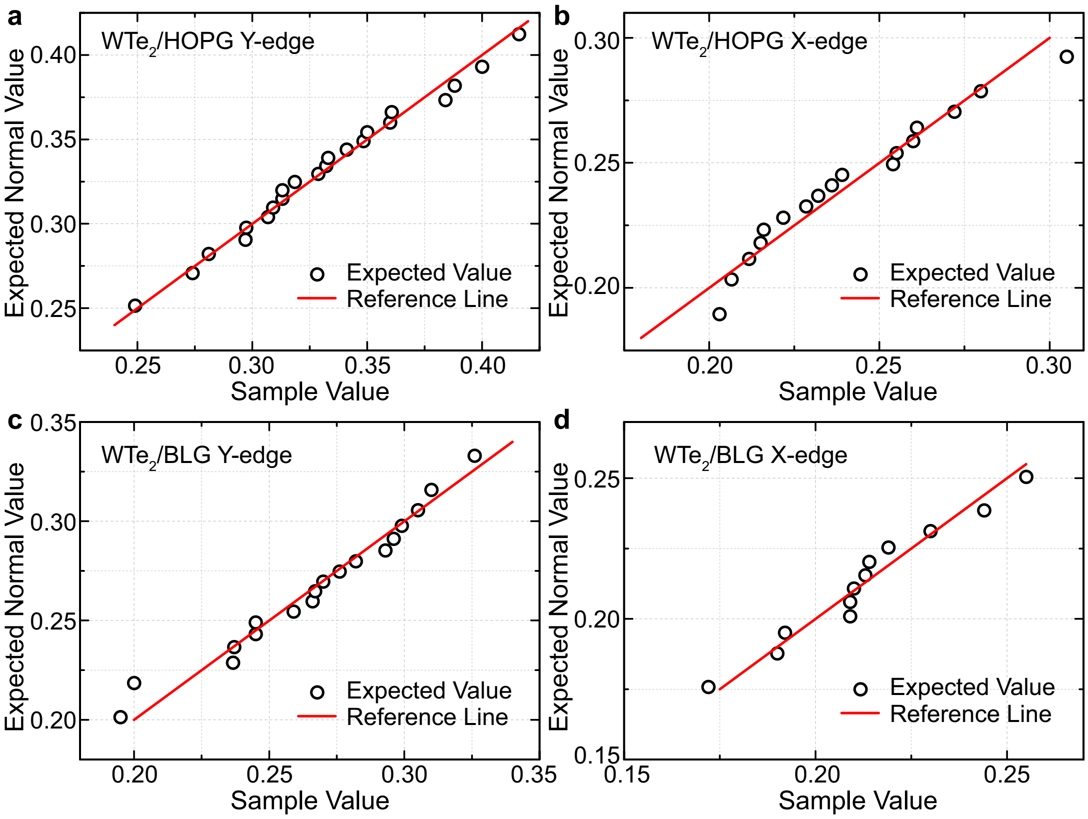

Supplementary Fig. 6: **Q-Q plots for the histograms in Fig. 3a,b in the main text.** We find all four data sets, when plotted against the expected values, lie on the identity line, confirming normality of the distributions. Slight deviations (skew) exists only for the X-edges of WTe_2/HOPG and WTe_2/BLG.

Given that the distributions are overlapping and that the sample number is relatively small, we further confirm that all distribution are statistically distinct. To this end, we run two-sample $T-$tests, applicable for small populations, with the null hypothesis that any pair of the four distributions are part of the same wider distribution. This procedure yields a p-value quantifying the statistical significance of the difference in mean.

Supplementary Table 1: Luttinger parameters K extracted from the histograms in Fig. 3a,b of the main text by fits to Gaussian normal distributions. The mean values of K, standard deviation σ, and standard error of the mean $SE_\sigma = \frac{\sigma}{\sqrt{n}}$, where n is the number of data points, are listed here and incorporated in Fig. 3c of the main text.

Edge Type / Substrate	K	σ	SE_σ
WTe_2/BLG X-edge	0.21	0.02	0.01
WTe_2/BLG Y-edge	0.27	0.04	0.01
WTe_2/HOPG X-edge	0.24	0.03	0.01
WTe_2/HOPG Y-edge	0.33	0.04	0.01

Supplementary Table 2: $p-$values obtained from two-sample $T-$tests on the distributions in Fig. 3a,b of the main text. The test returns $p = 1$ if any pair of distributions are identical, and $p = 0$ if they are perfectly distinct. For all pairs of distributions, we find $p < 0.05$, implying that the distributions are statistically distinct within a confidence interval of 95%.

Type \ Type	BLG X-edge	BLG Y-edge	HOPG X-edge	HOPG Y-edge
BLG X-edge	1	2.67×10^{-5}	7.20×10^{-3}	4.58×10^{-12}
BLG Y-edge	2.67×10^{-5}	1	2.35×10^{-2}	6.34×10^{-6}
HOPG X-edge	7.20×10^{-3}	2.35×10^{-2}	1	1.46×10^{-9}
HOPG Y-edge	4.58×10^{-12}	6.34×10^{-6}	1.46×10^{-9}	1

The $T-$value is defined as,

$$T = \frac{\mu_1 - \mu_2}{\sqrt{\frac{\sigma_1^2}{m} + \frac{\sigma_2^2}{n}}}, \tag{5}$$

where $\mu_{1,2}$ and $\sigma_{1,2}$ are the sample mean and standard deviation of distributions 1 and 2, respectively, while m and n are their number of samples. The degree of freedom df of the $T-$test (generally for unequal variances) represents the effective number of independent variables in the two distributions, defined as

$$df = \frac{(\frac{\mu_1^2}{m} + \frac{\mu_2^2}{n})^2}{\frac{\left(\frac{\mu_1^2}{m}\right)^2}{m-1} + \frac{\left(\frac{\mu_2^2}{n}\right)^2}{n-1}}. \tag{6}$$

Once the $T-$value and df are obtained, the $p-$value can be calculated as

$$p = 2 - 2 \times \mathrm{cdf}(T, df), \tag{7}$$

where cdf is the cumulative distribution function for the Student's $T-$distribution. Our $T-$test yields $p-$values $p < 0.05$ for any pair of two distributions (see Table 2), confirming that that each distribution is statistically distinct with a statistical significance of $> 95\%$.

S6. ELECTROSTATIC MODEL TO ESTIMATE THE LUTTINGER PARAMETER K

For a theoretical estimate of the Luttinger parameter K, we calculate the electrostatic energy stored inside a three-dimensional space-charge distribution along the sample edge. Such model was first proposed by Teo *et al* [5] and recently successfully employed by Stühler *et al* [6]. The charge density can be expressed as,

$$\rho_r(x, y, z) = \frac{Q}{w\xi L} e^{-x/\xi} \Theta(L/2 - |y|)\Theta(z)\Theta(w - z), \tag{8}$$

where L is the edge length, $w = 0.7$ nm is the vertical thickness of the charge distribution (which is given by the 1T'-WTe_2 monolayer thickness), and Q is the total charge. The charge distribution is concentrated along the sample edge-vacuum interface, and decays exponentially into the 2D bulk with a characteristic decay length $\xi \simeq 2$ nm (see Fig. 1 of the main text). To model dielectric screening by a substrate, we consider a substrate-dependent mirror

charge density at a vertical separation of 0.5 nm, corresponding to the van-der-Waals gap between the 1T'-WTe_2 monolayer and its substrate [7]. The image charge distribution is given by:

$$\rho'(x,y,z) = -\frac{\epsilon_r - 1}{\epsilon_r + 1}\rho_r(x,y,-z), \tag{9}$$

where ϵ_r is the dielectric constant of the substrate. The electrostatic energy stored in the edge state-image charge system is then evaluated numerically, via:

$$U_{\mathrm{E}} = \frac{1}{8\pi\epsilon_0}\int \mathrm{d}^3\mathbf{r} \int \mathrm{d}^3\mathbf{r}' \left(\frac{\rho_r(\mathbf{r})\rho_r(\mathbf{r}')}{|\mathbf{r}-\mathbf{r}'|} + \frac{\rho_r(\mathbf{r})\rho'(\mathbf{r}')}{|\mathbf{r}-\mathbf{r}'|} \right). \tag{10}$$

where $\mathbf{r}$, $\mathbf{r}'$ are coordinates of the individual point charges. The first term in Supplementary Equation(10) represents the unscreened interaction of real charges at the edge, while the second term represents the electrostatic interaction with the image charge. For an isotropic Coulomb interaction in the long-wavelength limit ($q = 0$), the Coulomb interaction strength W is equal to the potential $W = W(q = 0) = U_{\mathrm{E}}L$ [5, 6], which we then use to obtain K (Eq. 1 of the main text).

We note that our approach differs from that of Stühler *et al* [6], in that we evaluate Supplementary Equation(10) numerically rather than approximating it by a logarithmic interpolation between the two limits $\xi \ll w \ll L$ (i.e., negligible decay length) and $w \ll \xi \ll L$ (i.e., negligible channel width). Similar to bismuthene, the edge state decay length in 1T'-WTe_2 is comparable to the thickness of the atomic monolayer ($\xi \sim w$) [7, 8]. Hence, neither limit strictly applies to our samples. We find that a logarithmic interpolation of W, where:

$$W = \frac{e^2}{\pi\epsilon_0(\epsilon_r + 1)} \ln\left(e^{-\frac{1}{2}} 2^{-\epsilon_r}\frac{w}{L} + e^{1-\gamma}2^{-1}\frac{\xi}{L} \right) \tag{11}$$

provides reasonable agreement only at small ϵ_r (as in the case of Ref. [6]). However, this interpolation fails in the perfect metal limit ($\epsilon_r \to \infty$), where it predicts that $K \to 1$, which corresponds to the case of non-interacting electrons. Indeed, the finite interaction strength observed in our samples ($K \sim 0.35$), even in the metallic limit, is more accurately represented by a full numerical evaluation of Supplementary Equation(10), with the van-der-Waals gap between the sample and substrate taken into account. The agreement of this space-charge model with the experimental data is further confirmed by tight-binding calculations (see Fig. 3c of the main text and Sec. S7).

S7. FULL TIGHT-BINDING MODEL TO CALCULATE THE LUTTINGER PARAMETER K

The electronic structure in 1T'-WTe_2 was modeled by constructing a real space eight-band Hamiltonian for two Te and two W atoms in a rectangular unit cell. This low-energy Hamiltonian contains contribution from Te p_x orbitals and W $d_{x^2-y^2}$ orbitals, which reflects the distorted tetrahedral crystal structure of the monolayer [9–11]. Band structures from this model are shown in Fig. 3d,e of the main text for the Y- and X- directions of the Brillouin zone. In addition, we include the hopping matrix elements with the Rashba spin-orbit coupling terms. An example of a real-space tight-binding calculation of the LDOS for realistic edge disorder is shown in Supplementary Fig. 7, showing an STM topography image (a) of the same X-edge as in Fig. 1 and Supplementary Fig. 1, as well as with extracted roughness overlaid (e). Supplementary Fig. 7(b-d) and (f-h) compare measured dI/dV maps with calculated LDOS maps at energies indicated. The LDOS has been calculated for the particular atomic configuration at the edge as extracted / indicated in (e), and shows good agreement with the modulations observed in the experiments.

To obtain estimates for the Luttinger parameter, we construct a mean-field tight-binding Hamiltonian for 1T'-WTe_2, which has the following form:

$$H = \sum_{\mu,\nu,i,j,\sigma,\sigma'} t^{ij}_{\mu\nu\sigma\sigma'} c^\dagger_{i\mu\sigma} c_{j\nu\sigma'} + \mathrm{h.c.}, \tag{12}$$

where t is the hopping integral, indices i, j refer to the unit cell, $\mu, \nu = (1, 2, 3, 4)$ represent the atoms or orbitals within each unit cell, and h.c. stands for hermitian conjugate. The operators $c^\dagger_{i\mu\sigma}$ and $c_{j\nu\sigma'}$ are the creation and annihilation operators at a site with orbital and spin quantum numbers $\sigma = (\uparrow,\downarrow)$. Using the Bogoliubov-de Gennes (BdG) transformation, these operators can be written as:

$$c_{i\mu\sigma} = \sum_n (u^n_{i\mu\sigma}\gamma_{n\sigma} - \sigma v^{n\star}_{i\mu\sigma}\gamma^\dagger_{n\sigma}) \tag{13}$$

$$c^\dagger_{i\mu\sigma} = \sum_n (u^{n\star}_{i\mu\sigma}\gamma^\dagger_{n\sigma} - \sigma v^n_{i\mu\sigma}\gamma_{n\sigma}), \tag{14}$$

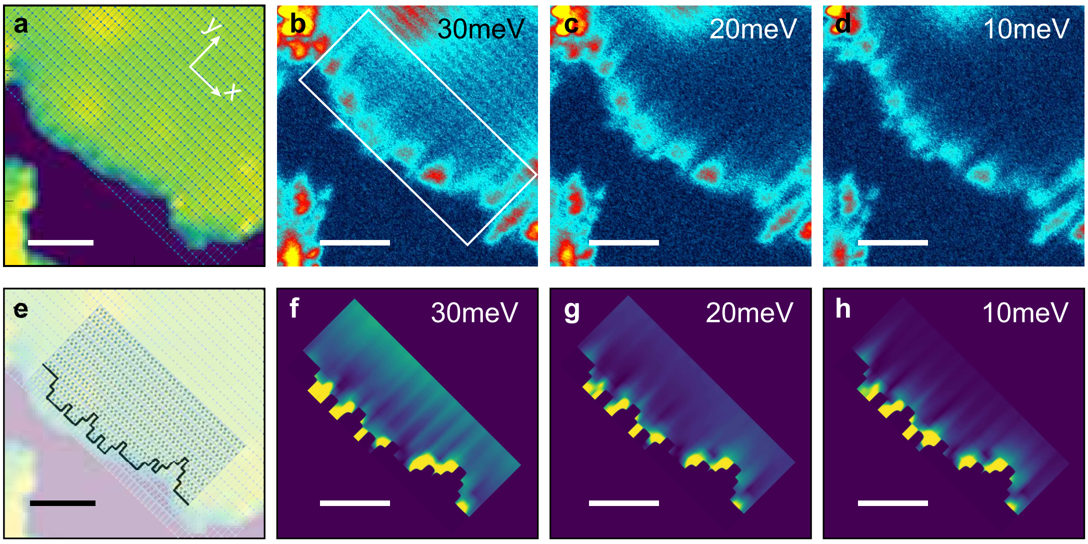

Supplementary Fig. 7: **Real-space tight-binding model of the WTe_2 X-edge in Fig. 1 of the main text and Supplementary Fig. 1.** **a**, Topography and **b-d**, $\mathrm{d}I/\mathrm{d}V$ map of the WTe_2 X-edge at energies indicated. **e**, Extraction of edge roughness from the STM topograph. **f-h**, Corresponding real-space tight-binding LDOS maps for the same energies as in **b-d**, which reproduce the observed modulations in the edge state's charge density well.

where u, v are the Bogoliubov coefficients and $\gamma, \gamma^\dagger$ are annihilation and creation operators.

Edges are created by applying open boundary conditions along the edge and periodic boundary conditions perpendicular to the edge. Diagonalising the above BdG Hamiltonian (Supplementary Equation(12) with the operators defined in Supplementary Equation (14)), we self-consistently calculate the electron density at each lattice site. The mean-field charge distribution $\rho_{i\mu\sigma}$ is given by:

$$\rho_{i\mu\sigma} = \sum_n |u^n_{i\mu\sigma}|^2 f(E_n), \tag{15}$$

where $f(E_n)$ is the Fermi-Dirac distribution at energy E_n. The mirror charge density ρ_m is then calculated using Eqs. 15 and 9 for $0 \leq \epsilon_r \leq 1000$, with a 0.5 nm separation between the substrate and WTe_2. By evaluating the U_E (Supplementary Equation (10)) using the charge densities from tight-binding calculations for different values of ϵ_r, we obtain K using Eq. 1 of the main text.

An important aspect to consider for the validity of our formalism is the assumption of $g_2 = g_4$ in the perturbative limit, where $g_{2,4}$ are density-density interaction terms, corresponding to the Hamiltonian contributions,

$$g_4(\psi^\dagger_{R\uparrow}\psi_{R\uparrow})^2 + g_2(\psi^\dagger_{R\uparrow}\psi_{R\uparrow}\psi^\dagger_{L\downarrow}\psi_{L\downarrow}), \tag{16}$$

where $\psi_{R\uparrow}$ annihilates a right mover electron with spin up, and $\psi_{L\downarrow}$ annihilates a left mover electron with spin down. We note that the assumption $g_2 = g_4$ is reasonable if the range of the Coulomb interaction is significantly extended. As shown in Supplementary Fig. 8, the Coulomb interaction has significant contributions up to the fifth nearest neighbour, owing to the finite width of the helical edge channel (note that the non-monotonous behaviour is due to the variation in electron density between the Te and W ions). We anticipate that our extraction of the Coulomb interaction strength may stimulate further in-depth theoretical treatment, for instance using density-matrix renormalization group (DMRG) methods.

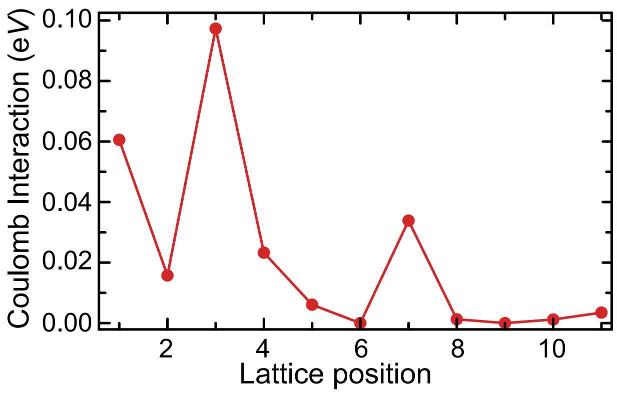

Supplementary Fig. 8: **Calculated Coulomb interaction as a function of the lattice position.** The Coulomb interaction has significant contributions up to the fifth nearest neighbour, corresponding to the fact that the helical edge channel has a finite width.

* Electronic address: b.weber@ntu.edu.sg

[1] Efros, A. L. & Shklovskii, B. I. Coulomb gap and low temperature conductivity of disordered systems. *J. Phys. C: Solid State Phys.* **8**, L49 (1975).
[2] Efros, A. L. Coulomb gap in disordered systems. *J. Phys. C: Solid State Phys.* **9**, 2021 (1976).
[3] Bartosch, L. & Kopietz, P. Zero bias anomaly in the density of states of low-dimensional metals. *Eur. Phys. J. B* **28**, 29–36 (2002).
[4] Altshuler, B. & Aronov, A. G. Electron-Electron Interactions in Disordered Systems, edited by A.L. Efros and M. Pollak. *(North-Holland, Amsterdam)* **1**, 155 (1985).
[5] Teo, J. C. & Kane, C. Critical behavior of a point contact in a quantum spin Hall insulator. *Phys. Rev. B* **79**, 235321 (2009).
[6] Stühler, R. *et al.* Tomonaga–Luttinger liquid in the edge channels of a quantum spin Hall insulator. *Nat. Phys.* **16**, 47–51 (2020).
[7] Jia, Z.-Y. *et al.* Direct visualization of a two-dimensional topological insulator in the single-layer 1T'-WTe_2. *Phys. Rev. B* **96**, 041108 (2017).
[8] Tang, S. *et al.* Quantum spin Hall state in monolayer 1T'-WTe_2. *Nat. Phys.* **13**, 683–687 (2017).
[9] Lau, A., Ray, R., Varjas, D. & Akhmerov, A. R. Influence of lattice termination on the edge states of the quantum spin Hall insulator monolayer 1T'-WTe_2. *Phys. Rev. Mater.* **3**, 054206 (2019).
[10] Muechler, L., Alexandradinata, A., Neupert, T. & Car, R. Topological Nonsymmorphic Metals from Band Inversion. *Phys. Rev. X* **6**, 041069 (2016).
[11] Choe, D.-H., Sung, H.-J. & Chang, K. J. Understanding topological phase transition in monolayer transition metal dichalcogenides. *Phys. Rev. B* **93**, 125109 (2016).
